# Supplementary figures and images for: The DREAM complex through its subunit Lin37 cooperates with Rb to initiate quiescence
Source: eLife. 2017 Sep 18;6:e26876. doi: 10.7554/eLife.26876 (PMC5602299; doi:10.7554/eLife.26876)

Figure 5-figure supplement 1

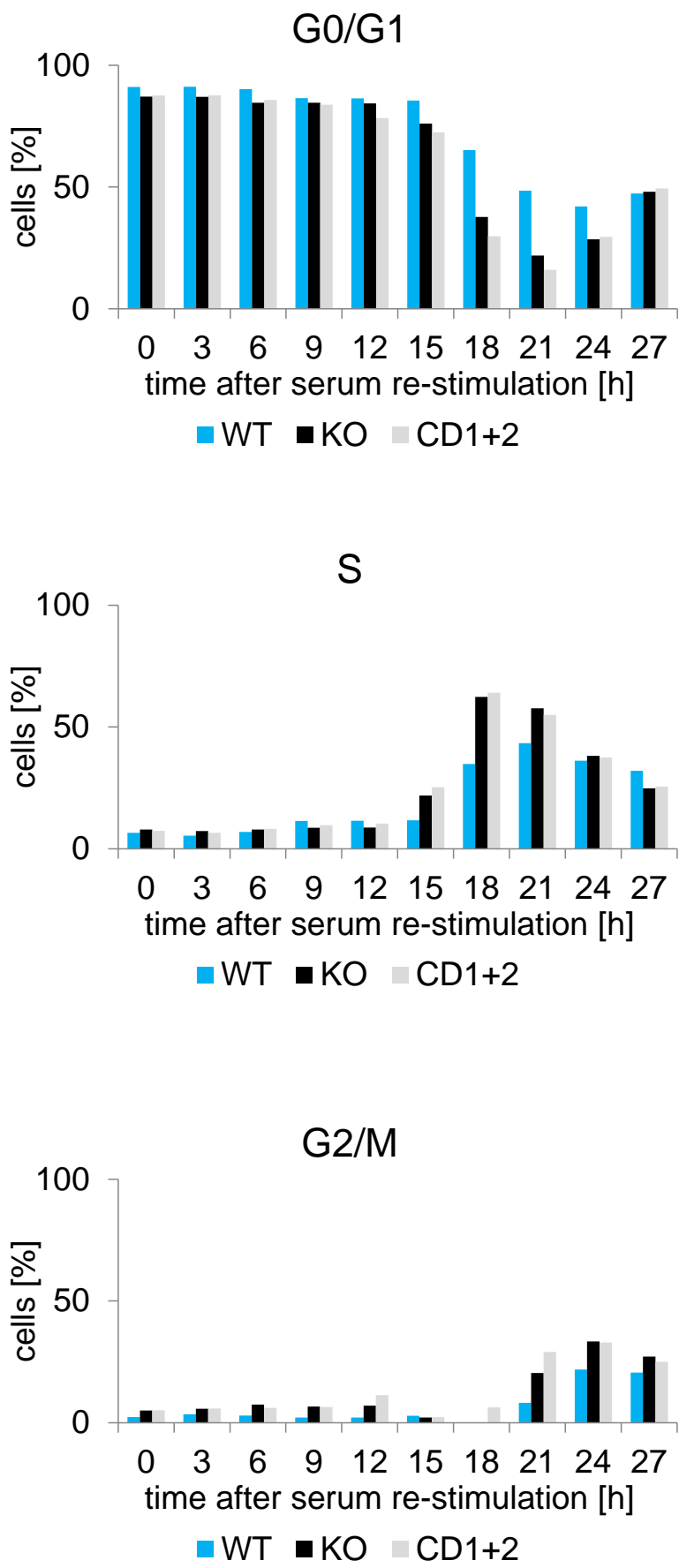

WT

KO

CD1+2

0h

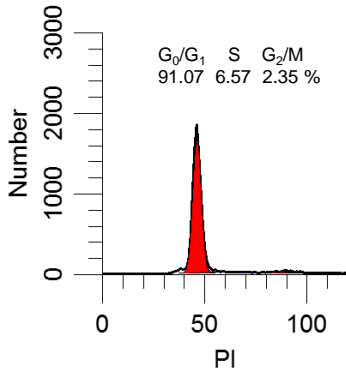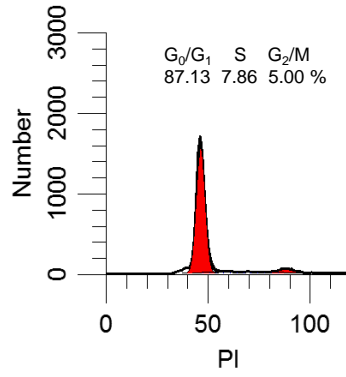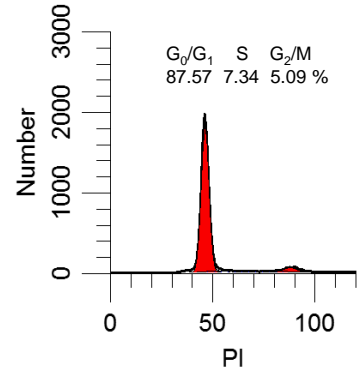

3h

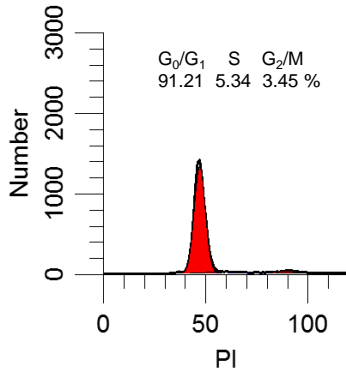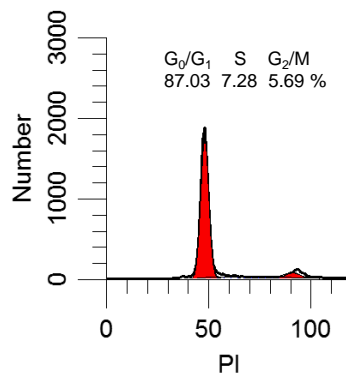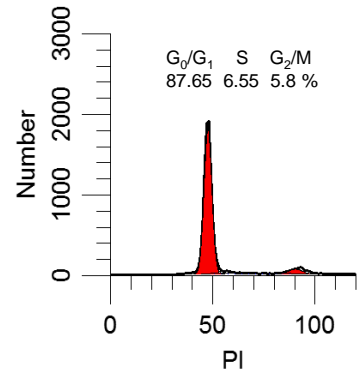

6h

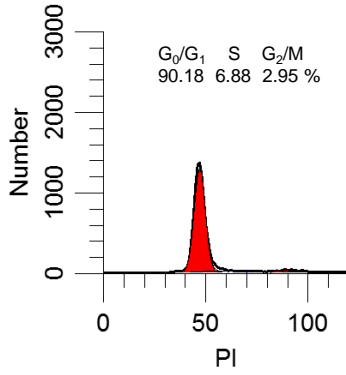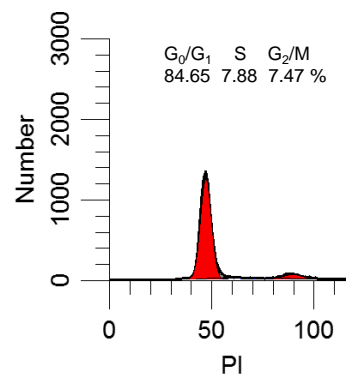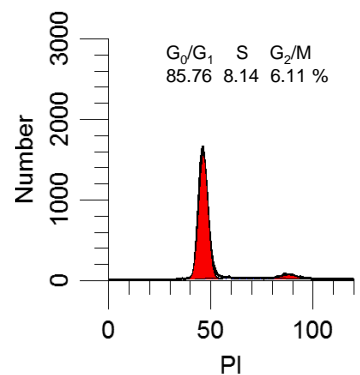

9h

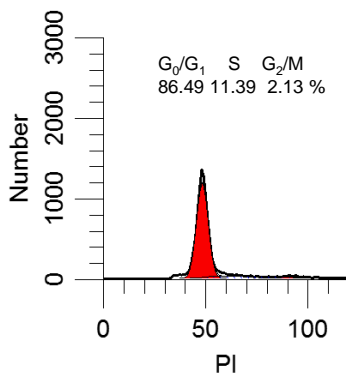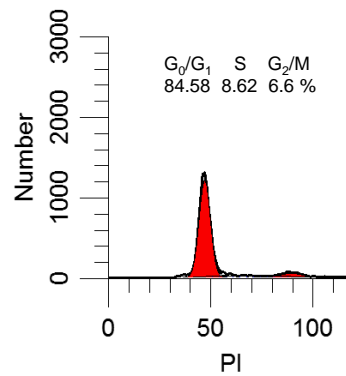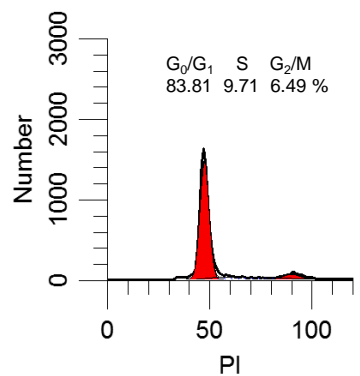

12h

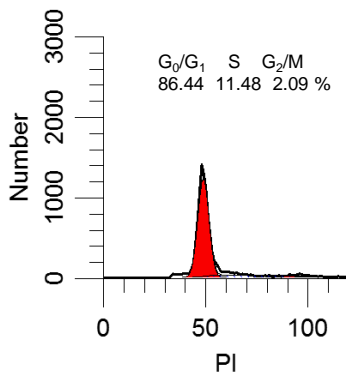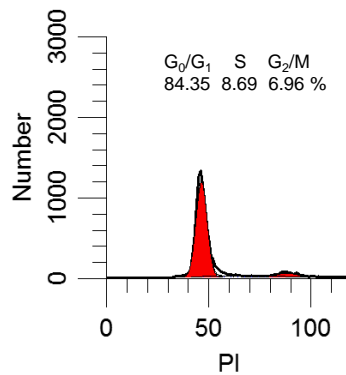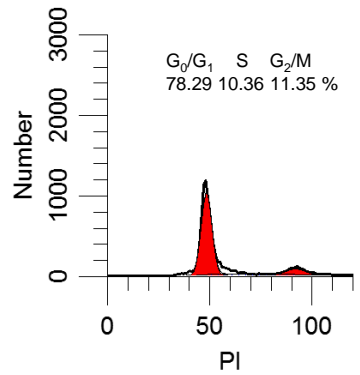

WT

KO

CD1+2

15h

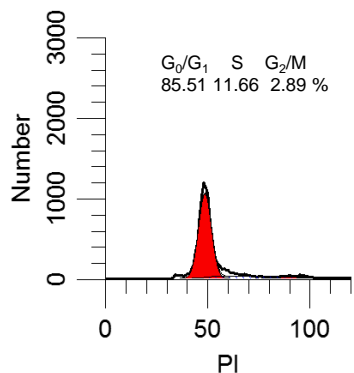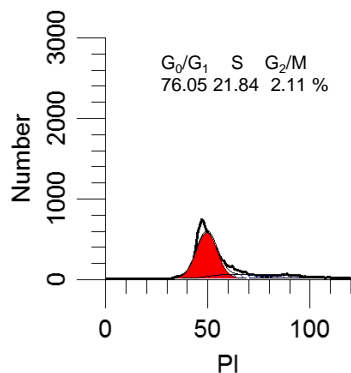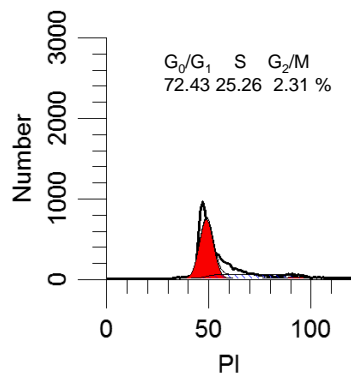

18h

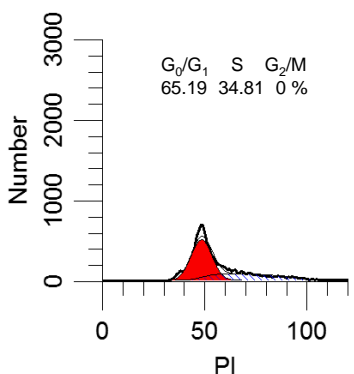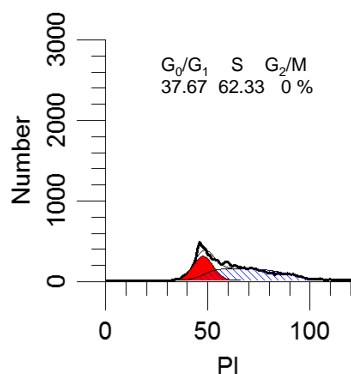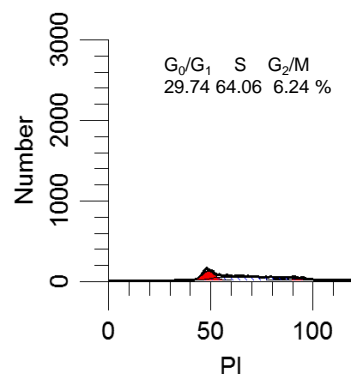

21h

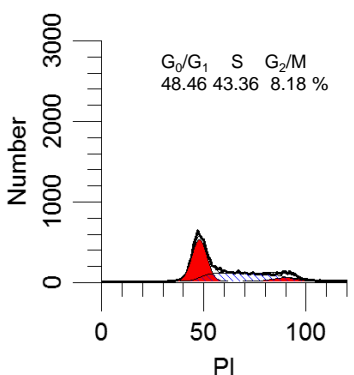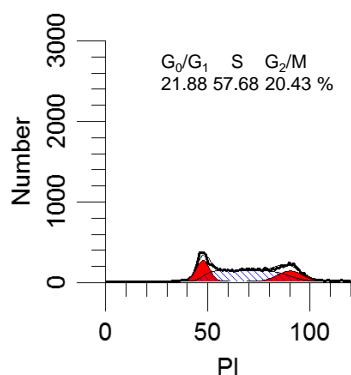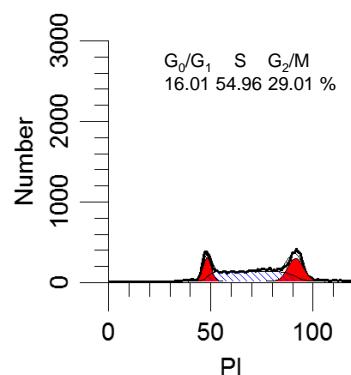

24h

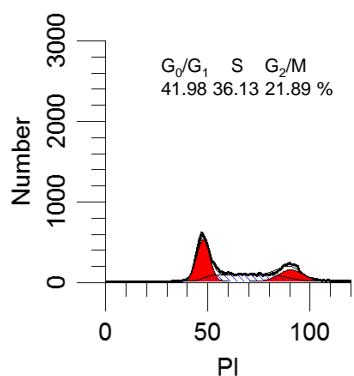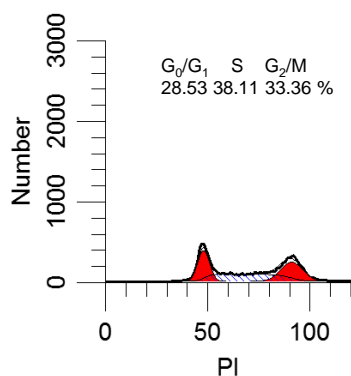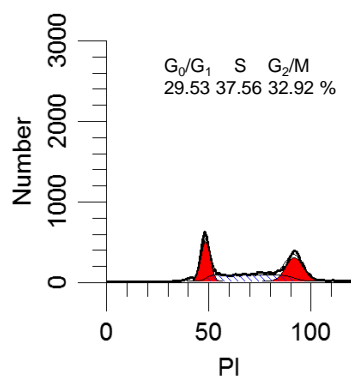

27h

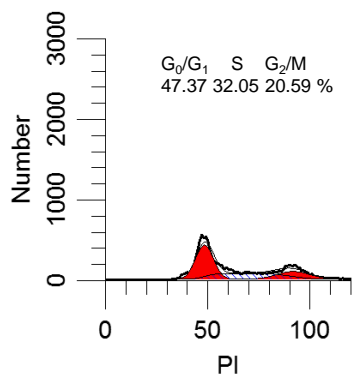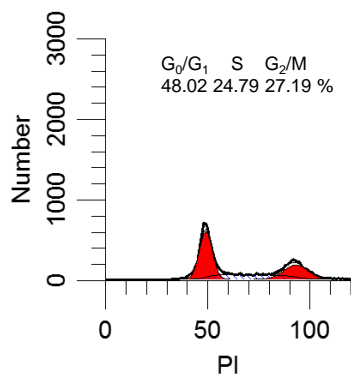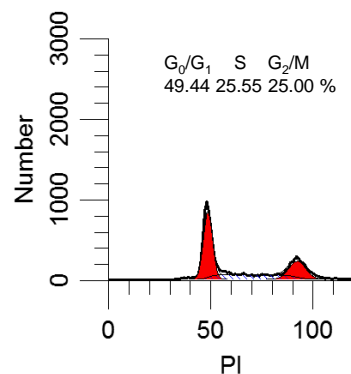

Supplement: Figure 5—source data 1. — Cells were arrested in G0 by serum starvation and re-entered the cell cycle after stimulation with 20% FCS. To determine cell cycle distribution of cell populations at specific time points after re-stimulation, DNA was stained with PI and fluorescence was measured by flow cytometry. (A) Percentages of cells in G0/G1, S, and G2/M at specific time points after re-stimulation. (B) DNA content as analyzed with ModFit LT 5.0. One representative experiment is shown. [file elife-26876-fig5-data1.pdf]
