# Supplementary material for: The DREAM complex through its subunit Lin37 cooperates with Rb to initiate quiescence
Source: eLife. 2017 Sep 18;6:e26876. doi: 10.7554/eLife.26876 (PMC5602299; doi:10.7554/eLife.26876)
Supplement: Figure 9—source data 1. — To determine cell cycle distribution of serum-starved and re-stimulated cell populations, DNA was stained with PI and fluorescence was measured by flow cytometry. (A) Percentages of cells in G0/G1, S, and G2/M at specific time points after re-stimulation. (B) DNA content as analyzed with ModFit LT 5.0. One representative experiment is shown. [file elife-26876-fig9-data1.pdf]

Figure 9-figure supplement 1

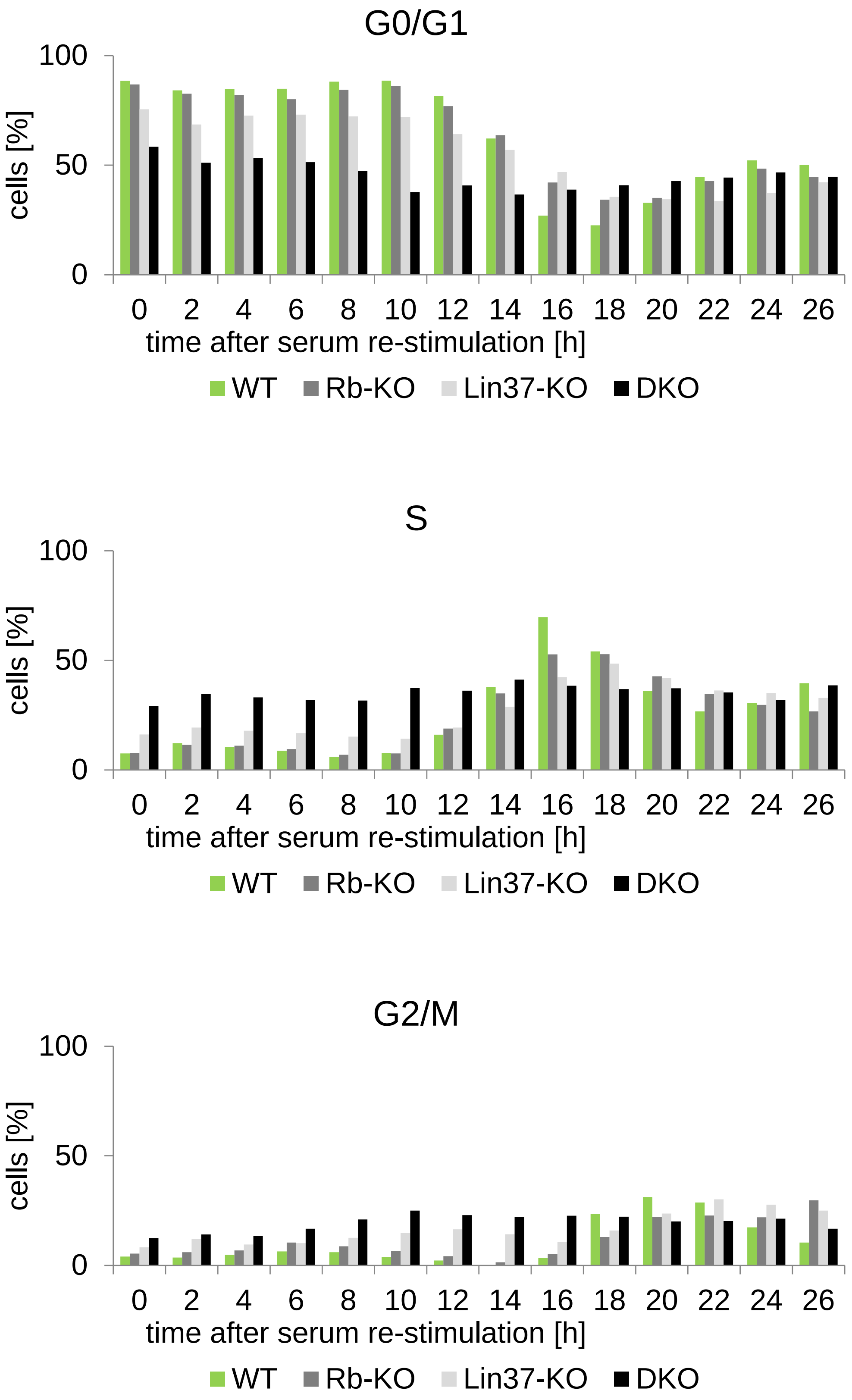

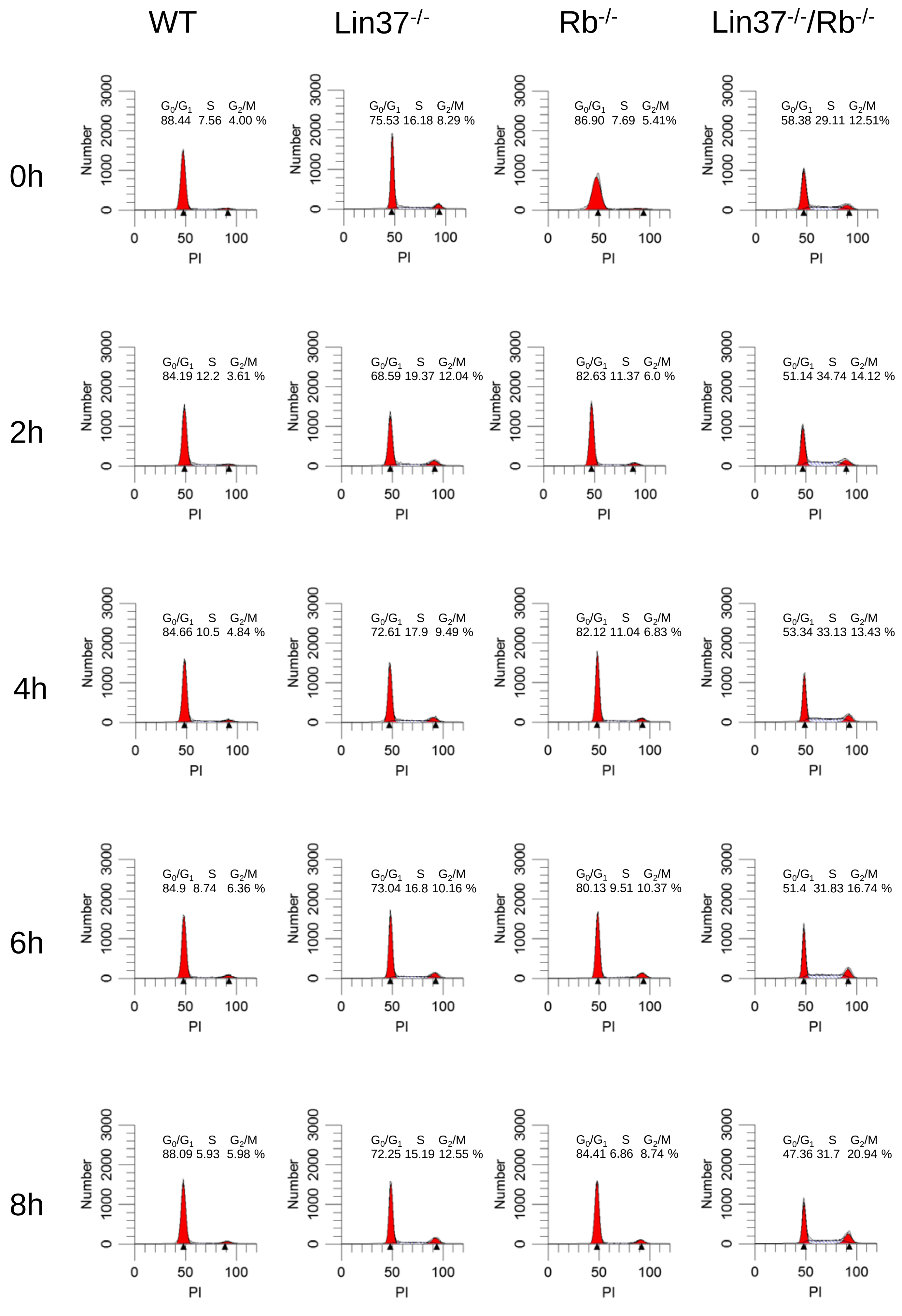

WT
Lin37$^{-/-}$
Rb$^{-/-}$
Lin37$^{-/-}$/Rb$^{-/-}$
0h
2h
4h
6h
8h
Number
PI
0 1000 2000 3000
0 50 100
G_0/G_1 S G_2/M
88.44 7.56 4.00 %
75.53 16.18 8.29 %
86.90 7.69 5.41%
58.38 29.11 12.51%
84.19 12.2 3.61 %
68.59 19.37 12.04 %
82.63 11.37 6.0 %
51.14 34.74 14.12 %
84.66 10.5 4.84 %
72.61 17.9 9.49 %
82.12 11.04 6.83 %
53.34 33.13 13.43 %
84.9 8.74 6.36 %
73.04 16.8 10.16 %
80.13 9.51 10.37 %
51.4 31.83 16.74 %
88.09 5.93 5.98 %
72.25 15.19 12.55 %
84.41 6.86 8.74 %
47.36 31.7 20.94 %

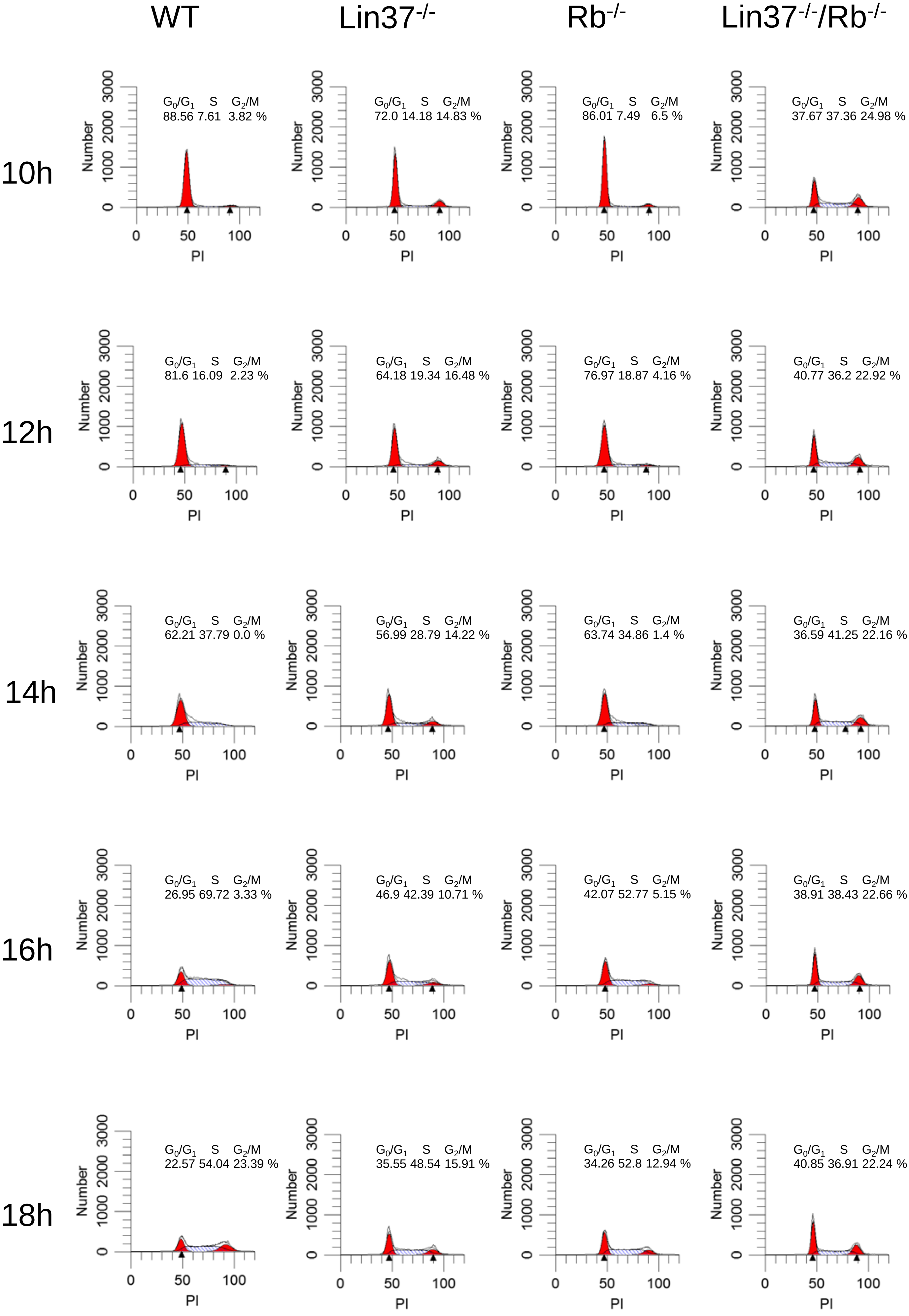
WT
Lin37-/-
Rb-/-
Lin37-/-/Rb-/-
10h
12h
14h
16h
18h
Number
PI
G0/G1 S G2/M
88.56 7.61 3.82 %
72.0 14.18 14.83 %
86.01 7.49 6.5 %
37.67 37.36 24.98 %
81.6 16.09 2.23 %
64.18 19.34 16.48 %
76.97 18.87 4.16 %
40.77 36.2 22.92 %
62.21 37.79 0.0 %
56.99 28.79 14.22 %
63.74 34.86 1.4 %
36.59 41.25 22.16 %
26.95 69.72 3.33 %
46.9 42.39 10.71 %
42.07 52.77 5.15 %
38.91 38.43 22.66 %
22.57 54.04 23.39 %
35.55 48.54 15.91 %
34.26 52.8 12.94 %
40.85 36.91 22.24 %

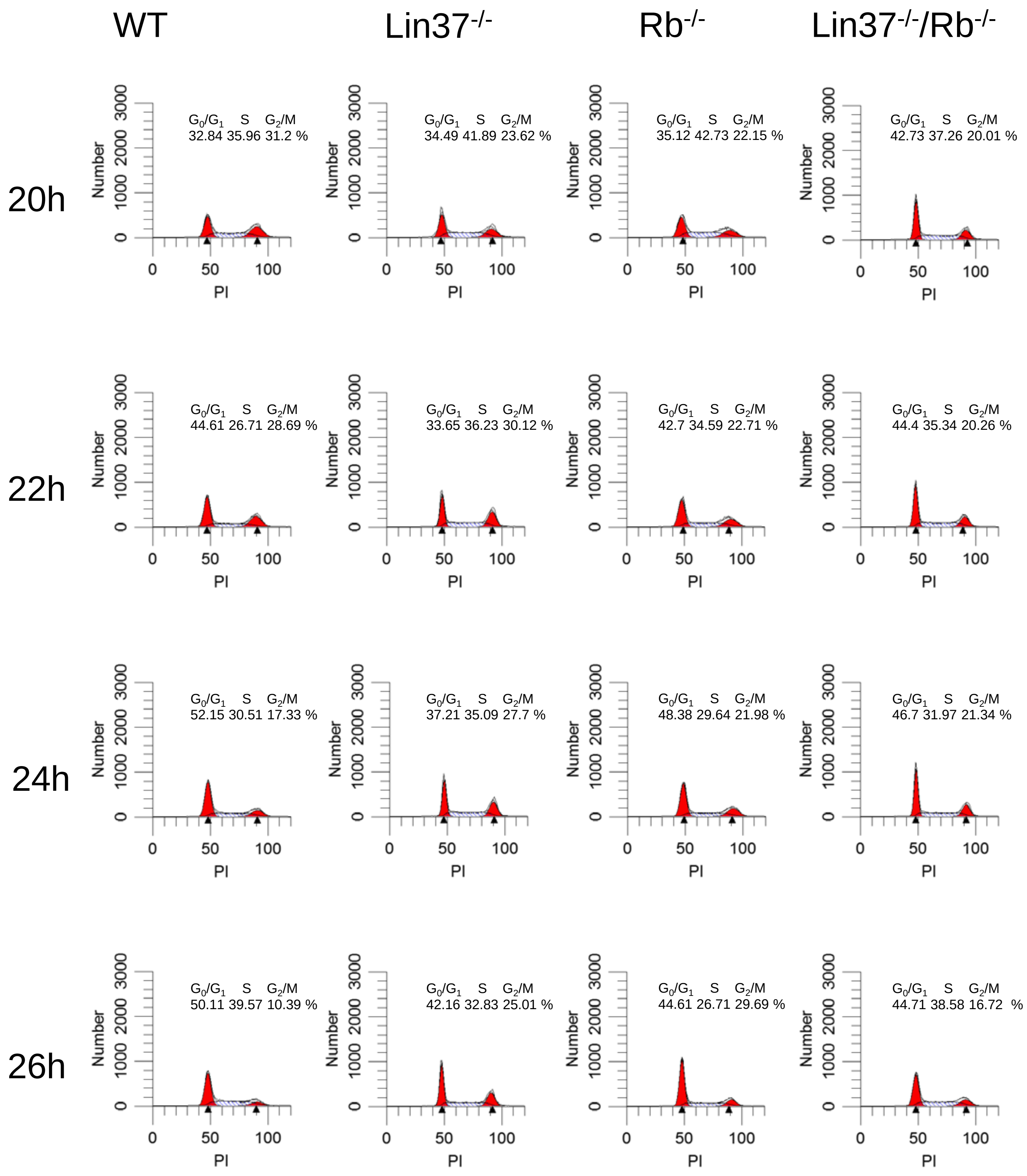
WT
Lin37-/-
Rb-/-
Lin37-/-/Rb-/-
20h
G0/G1 S G2/M
32.84 35.96 31.2 %
G0/G1 S G2/M
34.49 41.89 23.62 %
G0/G1 S G2/M
35.12 42.73 22.15 %
G0/G1 S G2/M
42.73 37.26 20.01 %
22h
G0/G1 S G2/M
44.61 26.71 28.69 %
G0/G1 S G2/M
33.65 36.23 30.12 %
G0/G1 S G2/M
42.7 34.59 22.71 %
G0/G1 S G2/M
44.4 35.34 20.26 %
24h
G0/G1 S G2/M
52.15 30.51 17.33 %
G0/G1 S G2/M
37.21 35.09 27.7 %
G0/G1 S G2/M
48.38 29.64 21.98 %
G0/G1 S G2/M
46.7 31.97 21.34 %
26h
G0/G1 S G2/M
50.11 39.57 10.39 %
G0/G1 S G2/M
42.16 32.83 25.01 %
G0/G1 S G2/M
44.61 26.71 29.69 %
G0/G1 S G2/M
44.71 38.58 16.72 %
Number
PI
0
50
100
1000
2000
3000
